# Self-Diagnosis of Mental Disorders: A Qualitative Study of Attitudes on Reddit

**DOI:** 10.1177/10497323241288785

**Published:** 2024-10-18

**Authors:** Rosie Underhill, Lucy Foulkes

**Affiliations:** 1 Department of Experimental Psychology, 6396 University of Oxford, Oxford, UK

**Keywords:** mental health, self-diagnosis, adolescents, social media

## Abstract

There is concern that a growing number of individuals, especially adolescents, are diagnosing themselves with mental disorders. However, there has been limited empirical research into this phenomenon: why it might happen, what the costs and benefits might be, and what the implications are for anyone who is experiencing distress. To address this, this study used reflexive thematic analysis to explore attitudes toward self-diagnosis of mental disorders as expressed on the discussion website Reddit. From 1195 user comments, five themes were generated: (1) *There is tension over who is the expert in diagnosis*; (2) *Self-diagnosis is a route to self-understanding in an inaccessible system*; (3) *Teenagers on social media are the problem*; (4) *Self-diagnosis can become self-fulfilling*, and (5) *Now no one is believed*. Together, these themes highlight that there is considerable anger, derision, and criticism targeted toward people who self-diagnose with mental disorders, and that this is particularly targeted toward adolescents who self-diagnose on or as a result of social media. The findings have important implications for understanding how to support and validate people, particularly adolescents, who (sometimes accurately) use diagnostic language to express how they are feeling.

## Introduction

Many individuals who experience mental health problems will seek advice from a medical professional, and this will sometimes lead to a diagnosis of a mental disorder. Other individuals who experience such symptoms will diagnose themselves, either before or instead of seeing a professional. This process of self-diagnosis typically begins online: individuals might investigate their symptoms via a search engine (a process dubbed “Dr. Google”), or find discussion of similar difficulties in online forums or on social media platforms such as TikTok (Farnood et al., 2020; Jutel, 2017; Milton et al., 2023). Through this process, they learn about a specific disorder, believe it is an accurate explanation of their symptoms or experiences, and begin using that diagnostic label to describe themselves.

Some individuals who self-diagnose with mental disorders will be correct: they are indeed experiencing the level of symptoms and functional impairment that would warrant a clinical diagnosis. In a recent study, participants were asked whether they had ever been professionally diagnosed with a number of mental disorders, and if not, whether they believed they should be (Rutter et al., 2023). Those who said they believed they should be diagnosed with depression or generalized anxiety disorder were experiencing a similar level of psychopathology (assessed via self-report questionnaires) to those with a professional diagnosis (Rutter et al., 2023). Nonetheless, there has been growing concern among academics and clinicians that adolescents, in particular, are increasingly diagnosing themselves with mental disorders they do not have (Acheson & Papadima, 2023; Haltigan et al., 2023). Thus, it is critical to understand the phenomenon of self-diagnosis of mental disorders: who self-diagnoses, why they do it, and what the benefits and costs might be.

Self-diagnosis of mental disorders likely has some benefits for the individual. A review of people’s perceptions of online self-diagnosis for physical health problems reported that self-diagnosis can offer a faster route to understanding one’s symptoms, compared to waiting a long time for a healthcare appointment (Farnood et al., 2020). Similarly, in countries where accessing health professionals is expensive, self-diagnosis is appealing and sometimes necessary due to financial constraints (Farnood et al., 2020). Participants also report that self-diagnosing ahead of a doctor’s appointment can help them feel better equipped during the clinical interaction, for example, by knowing what questions to ask and making use of limited time (Farnood et al., 2020). With regard to mental health problems specifically, there has been some speculation that self-diagnosis is useful because it gives the individual a culturally sanctioned framework for understanding themselves and their difficulties (Acheson & Papadima, 2023; Brinkmann, 2014); it is already established that diagnoses from professionals are helpful in this way (O’Connor et al., 2018). This is likely to be especially valuable in adolescence, since this is a period of crucial identity development in which individuals try to find language and concepts that describe and explain who they are (Branje, 2022); indeed, clinicians have suggested that self-diagnosis may offer a helpful means by which young people can understand and express their identity (Acheson & Papadima, 2023). Additionally, individuals may use their self-diagnosis to find resources on coping skills and to seek support from others with similar difficulties, much like those with a professional diagnosis (Lal et al., 2018).

Lastly, there is some speculation that adolescents benefit from self-diagnosis because it leads to attention from their peers (Dixon‐Ward & Chan, 2022; Teng et al., 2017). Adolescence is a period of heightened concern about peer approval and fitting in with friends (Tomova et al., 2021), and academics and clinicians have speculated that one motivation to self-diagnose might be to gain social attention and a sense of belonging (Corzine & Roy, 2024; David & Deeley, 2024; Fellowes, 2023). Specifically, the argument is that self-diagnosis garners sympathy and support from peers, or if other peers are self-diagnosing, it signals that they belong to a group with similar difficulties, both of which offer a crucial sense of social acceptance.

However, these assumptions are not necessarily held by adolescents themselves. Indeed, when attitudes were assessed among adolescents, there was recognition that self-diagnosis did lead to attention, but this was a bad thing; adolescents felt that peers who self-diagnose with depression are exaggerating or “faking it” for attention (Dixon‐Ward & Chan, 2022). In some cases, there was considerable disapproval toward the peers perceived to be doing this: for example, those with a professional diagnosis of depression reported that this form of “fake” self-diagnosis undermines the severity of their distress and increases public skepticism and stigma toward adolescent depression (Dixon‐Ward & Chan, 2022).

There are other proposals regarding why self-diagnosis of mental disorders may be problematic or harmful. First, there are concerns about a potential social contagion aspect of self-diagnosis, particularly online. Academics have speculated that some individuals might adopt a diagnostic label because they have been influenced by a friend or someone on social media doing the same (Haltigan et al., 2023), and there is evidence that people in online forums actively encourage others to use diagnostic language when interpreting symptoms (Giles & Newbold, 2011). On the one hand, this may lead some individuals to correctly identify and label their difficulties. However, on the other, some social settings might act as “incubators” for a form of self-labelling that is inconsistent with clinical criteria, particularly in the case of adolescents on social media (Haltigan et al., 2023). Interestingly, most discussion of this to date has focused on the latter, assuming that social contagion is erroneous and harmful rather than a useful means of individuals better understanding themselves; even the words “contagious” and “incubator” imply the problematic spreading of a disease. This aligns with a more general bias in the literature focusing on how adolescents might influence each other in a harmful, rather than helpful, manner (Foulkes et al., 2018; Molleman et al., 2022).

Second, for many decades, healthcare professionals have expressed concern that self-diagnosis (of any disorder) can make their job more difficult when they have to navigate conflicting opinions about the presenting symptoms (Jutel, 2017). Third, a number of academics and clinicians have written that self-diagnosis of mental disorders can lead people to unnecessarily adopt a “sick role,” cultivating a sense of vulnerability and removing people’s agency to cope with their distress and life’s typical challenges (Acheson & Papadima, 2023; Brinkmann, 2014). There is also concern that self-diagnosis can become a self-fulfilling prophecy, in which the individual begins to view all their experiences through the lens of the disorder, leading to changes in their self-concept and behavior that ultimately maintains or exacerbates symptoms (Foulkes & Andrews, 2023; Griffith & Stein, 2021; Werkhoven et al., 2022).

Thus, there appears to be a complex tension in attitudes toward self-diagnosis, in which it is recognized as being understandable and potentially beneficial to the individual, while also being the target of considerable skepticism and concern. To date, discussion about self-diagnosis of mental disorders has been largely theoretical; the empirical research we describe here has focused on the impact of professional diagnosis on individuals (O’Connor et al., 2018) or on self-diagnosis of physical health problems (Farnood et al., 2020). Concern about the self-diagnosis of mental disorders has been discussed in the mainstream media and in theoretical articles by academics and clinicians, but there has been limited empirical investigation of the general public’s attitudes toward and understanding of this phenomenon (Dixon‐Ward & Chan, 2022). However, these attitudes could offer vital insight into why people self-diagnose with mental disorders, how others respond to them when they do, and what the benefits and costs might be.

The current study seeks to contribute to this research area, by analyzing user comments in online discussions about self-diagnosis of mental disorders. The user comments are taken from Reddit, a discussion website with over 70 million daily users (Reddit, 2023). On Reddit, anonymous users post text, media, or external links, often posing a question, and other users then respond to the post using text and/or a voting system, in which the ratio of positive to negative votes determines how visible that content will be to other users. Reddit user comments (both the original post and subsequent responses) offer a rich source of data to examine societal attitudes toward a wide range of topics: there are over 100,000 active forums within Reddit, known as subreddits, each focusing on a specific subject (Reddit, 2023).

Using Reddit data to examine societal attitudes is advantageous for several reasons. First, its freeform nature allows individuals to express their attitudes in a way that is unrestricted by word limits or researcher–participant interactions (Rocha-Silva et al., 2023). Second, its scale means that attitudes from a large number of individuals can be analyzed. Third, the anonymity and online context likely encourages people to express themselves in a particularly candid way, in a phenomenon known as the “online disinhibition effect” (Suler, 2004). Given this, collecting Reddit data may garner particularly honest attitudes relating to self-diagnosis of mental disorders, including possibly contentious attitudes that individuals may not express when they can be personally identified.

Through thematic analysis of Reddit data, this study aimed to address the following overarching research question: “What are people’s attitudes toward the self-diagnosis of mental disorders, as expressed in online Reddit communities?” There were three sub-questions:

(i) What are the perceived benefits and harms of self-diagnosing with a mental disorder?
(ii) In what way might people have different attitudes toward self-diagnosis in adolescents compared to adults?
(iii) What factors are believed to contribute to someone self-diagnosing with a mental disorder?

## Method

### Ethical Considerations

Ethical approval was obtained through the authors’ divisional ethics committee. Ethical considerations were informed by existing guidelines for using internet-based data in research (Adams, 2024; Hewson & Buchanan, 2021; Markham & Buchanan, 2012). Informed consent was not obtained because Reddit users are anonymous, but this is widely considered acceptable because Reddit is a public website where there is likely to be no perception and/or expectation of privacy (British Psychological Society, 2021). Despite this, we took a number of steps to protect user anonymity and maintain ethical integrity. First, we collected data from publicly accessible, moderated subreddits with no statement prohibiting the use of their content for academic research. Second, in line with a similar previous study, we only included subreddits with a minimum of 1000 members, as users in these forums would likely consider posts within this context to be more public than private in nature, and we collected Reddit data that was at least 1 month old to give moderators time to identify and remove any personal information (Dixon‐Ward & Chan, 2022). Third, following existing guidance (McDermott et al., 2013), we cleaned the data by replacing usernames with participant numbers and removing dates of comments, subreddit links, and titles. Finally, we paraphrased quotes that appear in this paper with the aim of capturing the meaning of the comment without it being traceable to the original user via a search engine (Reagle, 2022). We entered all presented quotes into an online search engine and Reddit to confirm that we could not trace them back to their original source.

### Data Collection Procedure

Comments were obtained from three subreddits on relevant topics (unnamed to preserve anonymity) and a fourth search using the general Reddit search function. In each location, searches were conducted for the terms “self-diagnosis” and “self-diagnosing” individually. The Reddit search filter was set to display threads that were created within the past year. Threads were manually filtered based on the following inclusion criteria: (i) written in English, (ii) posted on moderated, public subreddits with a minimum of 1000 members, (iii) posted between July 2022 and June 2023 (i.e., in the 12 months preceding data collection), (iv) posted within a thread of at least five comments, and (v) discussed self-diagnosis in the context of “mental illness,” “mental health,” or a specified mental disorder (e.g., anxiety and dissociative identity disorder). For threads exceeding 100 comments, only the first 100 comments were analyzed (this number was selected for pragmatic reasons, to capture an appropriate depth and breadth of responses within and across threads without generating an unwieldy dataset; this kind of pragmatic decision regarding sample size is typical in large-scale qualitative research (Braun et al., 2021).

The exclusion criteria were as follows: (i) comments including an external link, article, picture, or video, (ii) comments with fewer than 10 words, (iii) comments from an automoderator (a Reddit bot that enforces community guidelines) or deleted user (N.B. if the deleted user created the original post in the thread, then only subsequent comments were analyzed), (iv) comments that discussed self-diagnosis only in relation to neurodevelopmental conditions (e.g., ADHD or autism) and/or physical health conditions (including functional neurological disorders and Tourette’s), and (v) comments from subreddits prohibiting the inclusion of its content in academic research. Comments related to neurodevelopmental conditions and physical health conditions were excluded due to conceptual reasons: it is likely that there are different factors associated with self-diagnosis for these individuals, particularly with regard to the benefit of understanding one’s long-term identity via the self-diagnosis of neurodevelopmental conditions, that require separate study (Overton et al., 2023). We therefore chose to explore only self-diagnosis of mental disorders in this study to keep our research questions sufficiently focused (Koro-Ljungberg & Hayes, 2010).

For each search, all threads were analyzed after taking into account the inclusion/exclusion criteria above. If there were more than 50 threads for a given search, only the first 50 threads were analyzed. This was again done for pragmatic reasons, in terms of achieving an appropriately rich dataset that was not unwieldy to analyze (Braun et al., 2021). This yielded 87 threads, with a total of 2247 user comments (a combination of first posts and subsequent responses). Using the RedditExtractoR package within R (Rivera, 2023), the data from these threads were scraped from Reddit’s application programming interface (API) into an Excel spreadsheet. For each thread, the number of scraped comments was manually checked against the number of original comments on Reddit to reduce the risk of missing data, which has been observed in previous studies that used similar computational methods to collect Reddit data (Gaffney & Matias, 2018). The scraped comments were then manually checked in line with the inclusion/exclusion criteria, and the entire dataset was cleaned to protect anonymity. The final dataset consisted of 87 threads with a combined total of 1195 comments and first posts from 813 contributors. The mean word count for all included comments and first posts was 83.55 words (*SD* = 116.25, range = 10–2367).

### Analysis Strategy

The data was analyzed using reflexive thematic analysis, a qualitative approach that enables researchers to identify shared patterns of meaning (e.g., understandings, perceptions, and views) within a dataset. Reflexive thematic analysis views researchers as active agents who bring their experiences, values, and mannerisms to the research process (Braun & Clarke, 2019; Varpio et al., 2017). When acknowledged by a reflexive researcher, these qualities promote rich and thoughtful engagement with the data (Terry et al., 2017).

We used a combined inductive–deductive approach, involving analysis that was primarily data-driven but partly guided by existing conceptual ideas regarding the self-diagnosis of mental disorders (Braun & Clarke, 2022; Byrne, 2022). Additionally, we took a critical realist stance to analysis. Critical realism describes the position that a person’s knowledge and how they express it is inevitably affected by and constructed through their language and socio-cultural context, but that there are also underlying structures and phenomena that exist in the outside world that shape their knowledge (Willig, 2008). Thus, we consider that a person’s experiences and opinions, including those shared in Reddit forums, are subjective but also grounded in real social and material circumstances (such as access to healthcare or exposure to social media). This critical realist stance has shaped our thematic analysis, in which we consider the wider social context as well as the specific experiences and attitudes expressed by users.

### Reflexivity Statement

Reflexivity involves examining how researchers’ experiences and values shape how knowledge is created from the data (Willig, 2008). Both authors have experienced chronic medically unexplained physical health symptoms and acknowledge that this has contributed to a sense of understanding as to why people might self-diagnose and an interest in its possible benefits and harms to the individual. Author 1 has also worked in a clinical health psychology service, where she became particularly interested in the impact that language use may have on a person’s well-being. Author 2’s research program focuses on understanding the recent increase in adolescent mental health problems, and she is particularly interested in understanding how increased mental health awareness, including on social media, has impacted individuals’ understanding of their mental health. Both authors acknowledge that these personal and professional experiences have likely shaped their interpretation of the data and informed the analysis presented here.

### Analytical Procedure

Reflexive thematic analysis was conducted, following Braun and Clarke’s six-phase approach (Braun & Clarke, 2006, 2019). NVivo version 14 was used to support the analysis (Lumivero, 2023). To support reflexivity, author 1 kept a reflective journal (see Supplemental materials 1). Author 1 led the analysis, but both authors engaged in crystallization together to deepen her understanding and interpretation of the data and wider research area, and to support credible findings (Ellingson, 2008; Tracy, 2010) (see Supplemental materials 1).

In the first phase defined by Braun and Clarke, *data familiarization*, author 1 read through the entire dataset during the cleaning process and noted possible patterns in her reflexive journal (see Supplemental materials 1; Braun & Clarke, 2006). To begin the second phase, *coding*, the authors initially independently coded the same randomly selected 10% of the dataset and discussed this together during a crystallization meeting. During this meeting, both authors shared early thoughts about potentially important ideas in the data. For example, it was discussed how “fake” self-diagnosis could be understood in two ways, either as being deliberately fake (i.e., pretending to have a mental disorder) or deluded (i.e., mistaking sub-clinical levels of distress as disorder) and how users typically did not clarify how they distinguished these two (see Supplemental materials 1). At the end of this crystallization process, author 1 had a list of 220 codes.

Building on these initial codes, author 1 then generated codes for the remainder of the dataset. Data was coded primarily at a semantic level (i.e., focused on explicit, surface-level meaning rather than possible underlying concepts or implied meaning). At the end of this phase, there were 450 codes. The authors then had a second crystallization meeting to discuss these 450 preliminary codes. After this meeting, author 1 carried out several steps to produce a more manageable number of codes for the next phase of analysis; similar codes were collated, duplicate codes were merged, and some codes were discarded because they were associated with shallow, sparse data. These steps reduced the 450 preliminary codes to a list of 90 codes (see Supplemental materials 2) and, based on these 90 codes, author 1 began the third phase of analysis, *theme generation*. Five preliminary themes were generated (see Supplemental materials 1).

During the fourth and fifth phases, *reviewing themes* and *defining and naming themes*, the authors had a third crystallization meeting. Discussion centered around the coherence of the themes in relation to the codes and the entire dataset, and selecting names that best reflected the narrative of each theme (see Supplemental materials 3). The five final themes are described in more detail below.

## Results

**Figure 1.**
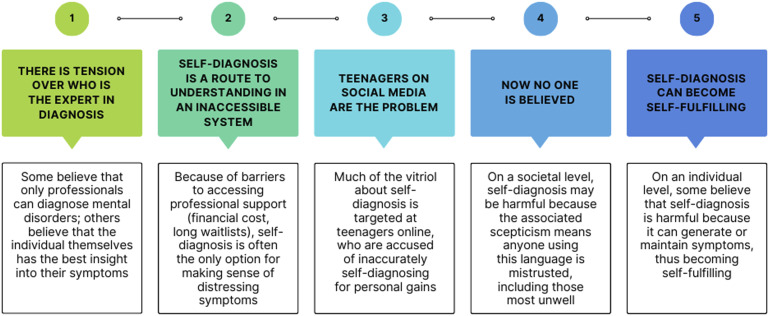
Schematic summary of the five themes.

The analysis produced five intersecting themes: (1) *There is tension over who is the expert in diagnosis*; (2) *Self-diagnosis is a route to self-understanding in an inaccessible system*; (3) *Teenagers on social media are the problem*; (4) *Self-diagnosis can become self-fulfilling*, and (5) *Now no one is believed*. Each theme is described in detail below (Figure 1).

### Theme 1: There is Tension Over Who is the Expert in Diagnosis

In these Reddit forums, there was considerable disagreement about who has the expertise to diagnose mental disorders: the professionally trained clinician or the person experiencing the symptoms. This led to conflicting opinions over the acceptability of self-diagnosis. Most commonly, people viewed clinicians as the experts in diagnosis, and therefore any attempts to self-diagnose were seen as inevitably inaccurate, flawed, or biased. For these individuals, clinicians were considered the only legitimate route to diagnosis because of the complex professional knowledge required:

It’s essential that clinicians diagnose. Diagnostic criteria need to be considered alongside lots of factors, including levels of distress and interference with daily life.

This complex professional expertise was contrasted with self-diagnosis via the internet, which was presented as sub-standard and rife with misinformation. The external opinion offered by clinicians was also considered an essential aspect of diagnosis: people reported that “even clinicians” should not self-diagnose because an outside perspective provides the necessary, objective opinion to mitigate bias. Indeed, people reported that self-diagnosis is susceptible to confirmation bias, where people interpret their symptoms in a way that confirms their existing beliefs:

You might find yourself interpreting behaviours as symptoms of the disorder you believe you have.

These individuals therefore argued that self-diagnosis was acceptable only as a transitory theory before clinical judgment was sought. This group argued that since clinicians are the experts in diagnosis, self-diagnosis should be discussed using tentative language only; for example, one user wrote, “It’s okay to say I think I have X, but you can’t say I have X until you have a clinical diagnosis.” Some people even argued that “self-speculation” and “self-suspecting” are more appropriate terms than self-diagnosis, implying that diagnosis is a protected, exclusive concept and that the power to diagnose resides in a clinician only.

In contrast, other Reddit users argued that the individual themselves has the necessary knowledge to self-diagnose and that therefore this process is entirely reasonable and acceptable. Indeed, several people argued that a clinical diagnosis of a mental disorder is based on an individual’s self-reported symptoms anyway, and that mental disorders are therefore “inherently self-diagnosed.” Since diagnostic criteria are widely available online, they believed that self-diagnosis is entirely logical and reasonable:

A doctor can’t look at you and say you are depressed. They can only ask you questions that you can already ask yourself.

Taking this further, some people argued that self-diagnosis is not only reasonable but also recommended. These users found it illogical that they would need a clinical diagnosis because they considered themselves to be the experts of their own experience and believed that they (and only they) have the self-insight required to make the most accurate diagnosis:

Simply read up on different disorders … you have the best understanding of yourself.

Another group acknowledged both sides of this tension and believed that ultimately clinicians and the individual themselves are both experts, both “useful pieces of the puzzle.” Given this, collaboration was believed to lead to the most accurate diagnosis. Ultimately, these users believed that while individuals can provide expert insight into their own experiences, this should be considered alongside clinical expertise for the most accurate diagnosis:

You have the most insight into yourself, but a clinician has the most insight into how to diagnose, so it makes sense to collaborate.

In summary, most users believed that diagnosis of mental disorders requires at least some clinical involvement and therefore self-diagnosis is acceptable only as a transitory theory before professional assessment. However, the disagreement from others (who believed that the necessary expertise lies in the individual experiencing symptoms) highlights that self-diagnosis is a contentious topic and that anyone attempting to self-diagnosis is likely to be met with at least some skepticism about whether their description is accurate or valid.

### Theme 2: Self-Diagnosis is a Route to Self-Understanding in an Inaccessible System

This theme highlights that some users felt self-diagnosis is understandable and unavoidable within the context of an inaccessible healthcare system. Two main barriers to accessing professional help were identified: high cost and long wait-times. Financial barriers were commonly discussed with reference to countries without government-funded healthcare systems, where individuals have to pay for healthcare insurance or services. People acknowledged that this can make getting a clinical assessment particularly challenging, and even impossible. More generally, long wait-times were also discussed; users commonly described having to wait several years until assessment. In light of these barriers, having the chance to be assessed and diagnosed by a clinician was viewed as a “privilege,” highlighting that while this is desirable, it is out of reach for many—and that self-diagnosis is therefore the only option for a person trying to understand and manage their distress:

Healthcare is incredibly hard to access for so many people, so if someone can recognise symptoms of for example depression, and use that to find coping strategies then that can be better than nothing.

Within this context, users acknowledged that self-diagnosis is understandable because it is challenging to live with uncertainty surrounding the nature of one’s own distress. Several users with a clinical diagnosis remembered this challenge and explained that, while waiting to see a clinician, self-diagnosis is often a desperate attempt to make sense of distress and gain some relief. However, even for these users, there was an overriding sense that self-diagnosis was acceptable only when there was no alternative, and ideally should happen only on a temporary basis. In line with Theme 1, users who were supportive toward self-diagnosis still said that ideally this should be a transitory step before finally getting professional help:

I was in a nightmare for years thinking there was something was wrong with me, until I found validation through self-diagnosis, before getting a clinical diagnosis.

### Theme 3: Teenagers on Social Media are the Problem

Individuals deemed to have self-diagnosed outside of these accepted boundaries were often heavily criticized by Reddit users. Theme 3 highlights that the most marked of this criticism tended to be targeted toward teenagers, particularly teenagers believed to have inappropriately self-diagnosed based on social media. Two types of inappropriate self-diagnosis were discussed: those who mistake low-level distress as disorder and those who intentionally deceive others by pretending to have a disorder. Although users made a distinction between these two groups, there was no detail about the process informing this.

With regard to the first type, users commented that low-level, transient distress is often being mistakenly self-diagnosed as a mental disorder (“People are confusing regular human emotions like stress or sadness with mental illnesses”). This type of inaccurate self-diagnosis was often discussed with frustration and anger.

Personality disorders are complex as f**k. I swear to God these kids think borderline personality disorder is having mood swings and a crush.

However, despite negative attitudes toward this behavior, there were conflicting attitudes toward whether this behavior is truly young people’s “fault.” There was recognition that this excessive self-diagnosis happens precisely because of young people’s developmental stage—specifically the emotional fluctuations associated with pubertal hormones and brain development in adolescence. Yet, identifying adolescent biology as the explanation for self-diagnosis also came with dismissive language—users commented that apparent symptoms are “probably just hormonal changes during puberty” or due to ongoing brain development. Thus, users seemed to use developmental explanations to be sympathetic and understanding toward teenagers who engage in inappropriate self-diagnosis, while in parallel using the same explanations to dismiss and disregard their distress.

Similarly, there was a recognition that while social media use contributes to inappropriate self-diagnosis in young people, this is not entirely their fault. Some users were clearly critical of how young people interpret the information they see online, particularly posts that encourage individuals to identify symptoms in themselves, with one user stating, “everyone under 30 thinks they can diagnose themselves now.” However, it was acknowledged that people make sense of their mental health using the language available to them—and misinformation on social media was believed to drive adolescents to misinterpret low-level distress as disorder.

Posts about mental health that acknowledge their difficulties … with vague or even incorrect information … social media blurred the line between struggles and illness.

There was also a sense that the combination of adolescent development and social media use makes young people particularly vulnerable to self-diagnosis. For example, some users suggested that ongoing brain development in adolescence makes it difficult for young people to understand the wealth of information online and the difference between transient distress and mental illness; others recognized that adolescents’ greater sensitivity to social influence makes them particularly likely to adopt the diagnostic language they see online. Again, the tone of posts seemed to indicate that while self-diagnosis may be understandable in light of misinformation on social media and the way this interacts with adolescent development, it is still likely to be inaccurate and therefore inappropriate.

Separately, users believed that some young people are engaging in a second form of inappropriate self-diagnosis: that of actively pretending to have mental disorders. This was identified by users explicitly writing that they thought people who self-diagnose were doing so intentionally, for example, by “lying for attention” or “role playing for fun.” This was typically viewed as an attempt to gain social status and establish an identity. Indeed, a wide range of terms were used to suggest that mental disorders have become attractive and socially desirable to some young people: “cool,” “trendy,” “quirky,” “special,” “a fashion accessory,” and “ticket into a club.” This form of inaccurate self-diagnosis was met with more explicitly negative attitudes. Some people held extremely strong negative opinions toward adolescents engaging in this practice, frequently swearing, implying that it is inexcusable:

They think that shit makes them quirky or interesting. It’s annoying as hell.

Much of this criticism was targeted toward dissociative identity disorder (DID). Specifically, users believed that adolescents may deliberately label themselves with DID as a way to explore different aspects of their identity through “roleplay” and gain peer validation. These motivations were typically linked to two adolescent-specific factors: identity development and increased sensitivity to peer pressure. Users believed that this behavior typically occurred within, and was motivated by, online communities, namely, TikTok and Discord. There was concern that this experimentation with identities had become a competition among groups of teenagers with individuals attempting to improve upon their peers’ diagnoses to gain social status:

There’s a huge competition amongst adolescents at the moment as to who has the most severe disorder. DID sits at the top.

In sum, users believed that adolescents in particular are often self-diagnosing in an inappropriate way and that social media and factors specific to adolescence contribute to this behavior.

### Theme 4: Self-Diagnosis can Become Self-Fulfilling

This theme explores the harmful impact that self-diagnosis of mental disorders may have on the individual who self-diagnoses. Typically, these attitudes were targeted toward individuals who *inaccurately* self-diagnosed (i.e., those who mislabelled milder forms of distress) but sometimes related to anyone who self-diagnosed. Users acknowledged that diagnostic language is powerful; the phrase “heavy label” was used implying that this language can have a significant influence over the way we perceive ourselves and our experience. Specifically, several users discussed the possibility that self-diagnosis can become a self-fulfilling prophecy, by triggering a change in thoughts, feelings, and/or behavior that ultimately increases distress or other symptoms. For example, some users wrote that self-diagnosis can “destroy” a person’s self-esteem, or can generate or exacerbate symptoms such as dissociation. One user referred to the damaging effect of self-diagnosis on their mental health as “auto-sabotage.” Additionally, an adolescent user (who gave their age in their comment) described their experience of pretending to have DID and outlines how the behavioral change that followed this had a wide-ranging negative impact on their mental health, education, and social life:

Over time, I stopped roleplaying characters everywhere and I genuinely believed I had DID. My social and academic life fell apart, and I felt empty.

Several users discussed how, once someone had adopted a self-diagnosis, the pathway to increased distress or other symptoms could happen very rapidly and was difficult to control. There was a sense that once someone had started down this path, sometimes described as a “rabbit hole,” it was almost impossible to turn back:

It’s so easy to inaccurately self-diagnose and this can have devastating long-term effects—you can unintentionally mindf**k yourself into having symptoms.

In sum, users highlighted that the language we use to understand our mental health has great importance; they believed that the way we define our experiences shapes our reality, and therefore self-diagnosing with a mental disorder can change an individual’s thoughts, feelings, and behavior in a destructive way that leads the diagnosis to become self-fulfilling.

### Theme 5: Now No One is Believed

Many users described how they have negative attitudes toward self-diagnosis because they believe it to be harmful for others. Specifically, users discussed how the “epidemic” of inaccurate self-diagnosis means that anyone claiming to have a mental disorder is now not being taken seriously. Users discussed how inaccurate self-diagnosis is misleadingly presenting mental disorders as either “cute or fun” or associated with transient, low-level distress, and this was seen to increase public misunderstanding of the severity of mental disorders by diluting or making light of it. The underlying assumption seemed to be that the increase in self-diagnosis was due to people wrongly labelling milder distress as a mental disorder and in turn shifting the public perception of mental disorder to the milder end of the symptom spectrum. Users with a clinical diagnosis described this misrepresentation as “exhausting,” “agitating,” and “horribly invalidating.” They highlighted that the “influx” of inaccurate self-diagnosis can make living with a mental disorder even more challenging and distressing, by undermining their own experiences:

People tend to dismiss my debilitating anxiety and depression because everyone has these disorders and they’re all fine.

In light of this misrepresentation, this group commented that it is hard to find support from others in a similar position because relevant online communities feel saturated with people identifying with disorders they do not have. As a result, users with clinical diagnoses described feeling misunderstood and invalidated even in spaces where they had hoped to find recognition and support.

However, the concern about being invalidated does not only involve those who are clinically diagnosed. Users also highlighted that self-diagnosing individuals are not being taken seriously, even when they should be. Indeed, some people seemed to assume that because self-diagnosis *can* be inaccurate, *every* self-diagnosis is inaccurate, which echoes attitudes highlighted in Theme 1. This generalization was conveyed through references to self-diagnosis being “completely invalid” and wrong in all cases. One user suggested that “all the self-diagnosers inevitably perform their disorders.” This group believed that since self-diagnosis is always wrong, self-diagnosis indicates that the person is a liar and that “the necessary response is social rejection.” In response to this, several users appeared to internalize others’ skepticism toward self-diagnosis, apologizing for appearing to self-diagnose and doubting the credibility of their own distress. In some cases, this prevented them from seeking professional support:

People dismissing self-diagnosis made me doubt myself. It took ages for me to feel brave enough to see a clinician because people kept saying “you aren’t anxious.”

This suggests that failing to take self-diagnosis seriously risks overlooking individuals who are struggling with their mental health, whether they have a mental disorder that is currently undiagnosed or are experiencing sub-clinical levels of distress that warrants support. Several users implied that even individuals who might be deliberately faking a mental disorder are all too often dismissed and that, while their self-diagnosis may be inaccurate, they too are often experiencing some level of distress that should be acknowledged and taken seriously. Thus, ironically, a consequence of increased self-diagnosis (i.e., an attempt to understand and communicate one’s distress) seems to be that many people in distress are now not being validated or believed. Ultimately, the narrative of this theme implies that psychiatric language should be reserved for those who really need it, but all levels of distress should be acknowledged and supported.

Together, the five themes highlight the complexity of the attitudes that people hold about self-diagnosis of mental disorder and contributed meaningful answers to our three research questions. With regard to our first question, about the perceived benefits and harms of self-diagnosis, our analysis indicated that self-diagnosis is considered to be both a powerful, helpful means of understanding and communicating one’s distress, and a risky practice that can lead to self-fulfilling prophecies and the undermining of other people’s difficulties. Our second question examined the possible differences in attitudes toward adults and adolescents who self-diagnose, and our analysis indicated that there were particularly aggressive, dismissive attitudes toward adolescents engaging in self-diagnosis, with the commonly held belief that they are self-diagnosing inappropriately due to a combination of factors related to social media and ongoing psychological development. Our third and final question, about what factors people believe lead to self-diagnosis, was answered across the analysis: Reddit users believed many factors are relevant, including practical necessity (lack of professional support), a desire to understand oneself, a need to cope, or a deceptive desire to gain attention from others. Ultimately, the attitudes presented here reveal a frequently hostile climate for anyone who attempts to diagnosis themselves with a mental disorder.

## Discussion

This study used reflexive thematic analysis to explore attitudes toward self-diagnosis of mental disorders, as expressed on the discussion website Reddit. This analysis highlighted that the self-diagnosis of mental disorders is a highly contested issue: while some people believe it is a helpful and understandable practice, others think it is inaccurate, performed for attention, and harmful both for those who self-diagnose and those who “really” have mental disorders. This anger and derision is particularly targeted toward adolescents who self-diagnose on or as a result of social media. The findings have important implications for understanding how to support and validate people who are experiencing symptoms of mental disorders and who use diagnostic language to express how they are feeling.

Across the dataset, there was a pervasive sense of moral judgment toward people who self-diagnose with mental disorders. A repeated message was that there are acceptable and unacceptable ways to self-diagnose and, in particular, that self-diagnosis is acceptable only as a temporary theory about oneself, which then needs to be discussed with a clinical professional to be validated. Anyone deemed to have engaged in unacceptable forms of self-diagnosis—which for some users was any self-diagnosis at all—was treated with vitriol and derision. Such negative attitudes toward self-diagnosis were often justified in light of its perceived harms, both to the individuals engaging in the practice (because the diagnosis could become self-fulfilling) and to society (because self-diagnosis devalues psychiatric terminology and leads to skepticism toward any admission of distress). In particular, there was a commonly held sense that people who self-diagnose misunderstand what mental disorders are and are doing a disservice to those who truly have clinical disorders because those who self-diagnose misleadingly send the message that mental disorders are mild. Users stated that people self-diagnose with mental disorders for attention or to experiment with a new identity, and are often knowingly being deceptive. Comments mocking or dismissing young people who self-diagnose were common.

In contrast, some Reddit users expressed sympathy toward those who self-diagnose with mental disorders: some argued that financial barriers or waitlists make clinical diagnosis almost impossible, and that labelling one’s symptoms with diagnostic language is therefore understandable as a means to make sense of and manage one’s distress. This aligns with existing evidence that self-diagnosis of physical health conditions is deemed acceptable when healthcare is inaccessible, and collectively this highlights how broad socio-economic issues impact public attitudes toward self-diagnosis (Farnood et al., 2020).

Additionally, this study extends previous literature by identifying a philosophical defense of self-diagnosis that may be specific to mental disorders. Some users stated that, unlike the objective assessments often used for diagnosing physical health conditions, professional diagnoses of mental disorders often rely on self-reported symptom measures, and thus the person who can best diagnose symptoms of mental disorders is the individual experiencing them. Even so, most users tended to revere professional diagnosis, treating it as an objective truth that was the ideal, preferred option, even if it wasn’t practically possible. Indeed, professional diagnoses were deemed so desirable in some cases that there was a sense of policing them; that users did not want these labels to be weakened or debased by being inaccurately co-opted by people with milder symptoms. This sense of ownership over psychiatric diagnosis has been described as “diagnostic possessiveness,” in which individuals who consider themselves more legitimate claimants of a diagnosis undermine the diagnostic claims of others (Lane, 2024). This possessiveness implies that the individuals involved believe psychiatric diagnoses to be “real” constructs, objective truths, which hold at least some power and value to them; in turn, they do not want these labels to be diluted or changed by others.

This belief and trust in the concept of psychiatric diagnosis is not universal. Indeed, the revering of diagnosis in this dataset is somewhat surprising, given that there has been much written about how psychiatric diagnoses are not objective entities with external boundaries but rather subjective constructs that may sometimes have nefarious aims or consequences—this discourse is typically referred to as the “anti-psychiatry” or “critical psychiatry” movement (Fountoulakis, 2022; Johnstone, 2018; Middleton & Moncrieff, 2019). Within this movement, there is concern that psychiatric diagnosis (and psychiatry as a discipline) affords too much power to clinicians and removes power from the individuals who are suffering (Moncrieff, 2010). However, we did not find evidence of this perspective in this dataset; instead, defenders of self-diagnosis elevated self-diagnosis rather than criticizing professional diagnosis. The more general validity of diagnosis as a concept was rarely disputed. Ultimately, there was a powerful tension within the dataset regarding who has the “right” to decide that a person has a mental disorder or to determine how a person should express their experiences, and there were impassioned moral arguments on all sides.

There was a similar tension with regard to whether self-diagnosis should be treated as accurate and understandable, or met with skepticism and dismissal. In line with existing literature, users in this study believed that adolescents in particular are self-diagnosing incorrectly, either by mislabelling mild distress or typical developmental stress as a mental disorder, or by deliberately faking a mental disorder in attempt to gain popularity or to experiment with a new identity (Acheson & Papadima, 2023; Dixon‐Ward & Chan, 2022; Teng et al., 2017). This attitude of disbelieving adolescents aligns with a long history of adolescents being dismissed or undermined when reporting their own experience and preferences (Baltag et al., 2022; Brierley & Larcher, 2016), including in the context of mental healthcare (Bergen et al., 2022; Hayes et al., 2019).

This criticism stands in interesting conflict with the belief, often given by academics writing on this topic, that self-diagnosis offers a ticket to social acceptance and belonging (Corzine & Roy, 2024; Giedinghagen, 2023). In contrast, the user comments in the current study indicate that self-diagnosing is perhaps more likely to be met with ostracism. We speculate that self-diagnosis might elicit both reactions, across different adolescents but perhaps also within the same adolescent in different contexts (e.g., an individual might find that a self-diagnosis garners them attention and validation on social media but derision among their real-life peers, or vice versa); these multifaceted responses to self-diagnosis is a key avenue for future research.

Our findings extend existing research by highlighting why people believe adolescents are particularly at risk. Notably, as shown in our (second) research question, we had hoped that users might specifically compare self-diagnosis in adults to that in adolescents. Instead, they focused on adolescence, so this only indirectly gives us clues about attitudes toward the two different age groups. Specifically, users speculated that certain aspects of social media (misinformation, the formation of “sick role” subcultures) interact with adolescence-specific factors (a time of identity development and heightened sensitivity to peer pressure) in a way that leads to inaccurate self-diagnosis. While this led some users to sympathize with adolescents who self-diagnose, others adopted a derisory, dismissive tone, implying adolescents were incapable of understanding themselves because of how social media interacted with their immature, developing brains. The implication was that adults are more capable of accurate self-diagnosis, but this was not stated explicitly in this dataset. Future research could better delineate attitudes toward self-diagnosis in adolescents compared to adults, using methodology such as interviews that directly ask participants planned questions.

These findings have important implications for understanding how to support people who self-diagnose with mental disorders, particularly young people. Some of them will be experiencing sufficiently severe symptoms to meet the diagnostic threshold for a disorder, but even those who do not are experiencing distress that needs to be understood (Acheson & Papadima, 2023). The current findings suggest that those who use diagnostic language to do so are at risk of being criticized or dismissed. This is likely to exacerbate the very distress they are trying to communicate and, based on users’ accounts, may even prevent some people from communicating their distress and delay any attempt to seek professional support. Thus, this study highlights an urgent need to identify strategies that ensure young people in distress are heard and understood. One possibility is that professionals and parents are encouraged (e.g., via public health campaigns) to take more seriously the use of self-diagnostic language in young people.

It will also be important to understand clinicians’ perspectives on and experience of self-diagnosis in patients, particularly adolescents. The limited literature to date indicates that some clinicians are critical of self-diagnosis and find it to be a hindrance to the treatment process and problematic to society, but they can also be understanding of the practice (Acheson & Papadima, 2023; Farnood et al., 2020; Jutel, 2017). Whatever the clinician’s views, it may be that self-diagnosis is an integral way that a young person understands their distress and it could perhaps be seen as a resource that both parties can use to better understand the young person’s difficulties. Once more research on self-diagnosis has been produced, guidelines could be developed for clinicians on how to understand and work with self-diagnosis in clinical practice. It would be helpful to co-produce these guidelines with adolescents themselves (Pavarini et al., 2019). The moral judgments toward self-diagnosis seen in this dataset are sometimes at odds with the increasingly recognized notion that adolescents are experts of their own experience, including their mental health problems, and that professionals should listen to them to better understand how to support them (Fusar-Poli et al., 2024). To address the tension of moral judgments toward those who self-diagnose, the voices and viewpoints of those with lived experiences should be heard when deciding how to best respond to (or make use of) self-diagnosis in clinical practice.

However, any expansion of self-diagnosis language potentially has a disadvantage that was highlighted repeatedly in the dataset: that if everyone self-diagnoses, it is difficult for the people suffering the most to be recognized. Our findings echo existing concerns from academics and clinicians that applying diagnostic language to mild forms of distress might be actively unhelpful and even harmful to some individuals by becoming self-fulfilling (i.e., generating or exacerbating symptoms) (Acheson & Papadima, 2023; Foulkes & Andrews, 2023). An alternative would be to find different frameworks that do not use diagnostic language but that nonetheless allow young people to understand and express their feelings and to be heard, validated, and understood. For example, alternative frameworks might include philosophical approaches, such as stoicism and existentialism, or biological approaches that explore neurocognitive development in adolescence. Future research is needed to understand which frameworks, including one that uses diagnostic language, are most useful for young people and the adults around them and which are most likely to lead to improved mental health outcomes. Given that self-diagnosis primarily occurs in response to (mis)information online, such frameworks could be used to develop publicly accessible online resources that support adolescents and adults to understand symptoms of mental disorders and broader psychological experiences.

Several limitations to the current research should be noted. First, the advantage of using Reddit data—that it allows researchers to understand what people think and feel outside of the context of being identifiable—can also be a disadvantage. We do not know the demographics of the users who contributed to this study, but in general, Reddit users are more likely to be male and under 49 years of age, and these characteristics may have impacted the resulting attitudes toward self-diagnosis (Pew Research Center, 2024). In addition, only 22% of the adult population use Reddit, meaning the views analyzed here are not necessarily representative of the population as a whole (Pew Research Center, 2024). Because of the format, we were also unable to plan the questions they were asked. In addition, the insights offered by this dataset were primarily about attitudes toward others self-diagnosing rather than insights from individuals who self-diagnose. Thus, this study should be complemented by future research that examines how self-diagnosis of mental disorders varies according to demographic characteristics such as gender and age, and the experience of people who engage in this practice. For instance, users made considerable reference to the idea that adolescents who self-diagnose often do so inappropriately. However, within existing research, there has been extremely limited understanding of how accurate self-diagnosis is or insight from young people themselves about why they might engage in this practice. Such insight is critical to determine the most effective ways of supporting young people to understand and communicate their distress.

To conclude, the current study provides a novel, in-depth exploration into attitudes toward those who self-diagnose with mental disorders. The findings highlight that this is a deeply contentious practice, with individuals stating emotional, moral arguments both for and against self-diagnosis. Many of these arguments centered around perceived benefits and harms of this practice, both for the individual who self-diagnoses and society. Much of the criticism toward those who self-diagnose with mental disorders was targeted toward young people, particularly those who self-diagnose on or as a result of social media. The findings have important implications for understanding how to support young people who use diagnostic language to communicate their distress, which should be built with further research.

## Supplemental Material

Supplemental Material - Self-Diagnosis of Mental Disorders: A Qualitative Study of Attitudes on Reddit Supplemental Material for Self-Diagnosis of Mental Disorders: A Qualitative Study of Attitudes on Reddit by Rosie Underhill and Lucy Foulkes in Qualitative Health Research

Supplemental Material - Self-Diagnosis of Mental Disorders: A Qualitative Study of Attitudes on Reddit Supplemental Material for Self-Diagnosis of Mental Disorders: A Qualitative Study of Attitudes on Reddit by Rosie Underhill and Lucy Foulkes in Qualitative Health Research

Supplemental Material - Self-Diagnosis of Mental Disorders: A Qualitative Study of Attitudes on Reddit Supplemental Material for Self-Diagnosis of Mental Disorders: A Qualitative Study of Attitudes on Reddit by Rosie Underhill and Lucy Foulkes in Qualitative Health Research
