# Supplementary material for: Self-Diagnosis of Mental Disorders: A Qualitative Study of Attitudes on Reddit
Source: Qual Health Res. 2024 Oct 18;35(7):779–92. doi: 10.1177/10497323241288785 (PMC12056264; doi:10.1177/10497323241288785)
Supplement: Supplemental Material - Self-Diagnosis of Mental Disorders: A Qualitative Study of Attitudes on Reddit [file sj-pdf-1-qhr-10.1177_10497323241288785.pdf]

Supplementary materials 1: Extracts from RU's reflective journal

31.07.2023: Data Familiarisation

Whilst cleaning the dataset, I engaged in the first phase of analysis: data familiarisation. This involved reading through the entire dataset and noting down any initial impressions and analytical ideas.

My initial impression of the dataset seems to be more feeling-based than thought-based – I feel a lingering sense of heaviness and sadness. The general consensus (so far) appears to be that people are increasingly self-diagnosing with mental disorders in an inaccurate way, and that this is harmful to those who are most unwell (by devaluing diagnostic language). In particular, Reddit users seem to believe that mental disorders are increasingly being glamourised and viewed as appealing/'trendy', especially by young people. Clearly, this is a distorted view of mental disorder and seems horribly ironic in light of increased awareness efforts around mental health problems. There seems to be a particularly strong sense that social media is driving people to inaccurately identify with mental disorders.

In parallel, I get the sense that most of the Reddit users who themselves are considering self-diagnosis don't actually want to self-diagnose. Many seem desperate for a professional assessment/diagnosis but accessing this is challenging. Given this, self-diagnosis seemed to be their only option for trying to understand their distress – diagnosing themselves didn't feel ideal, but it felt necessary within the context of an inaccessible healthcare system. This is interesting from a critical realist perspective - it highlights how our broader social context (i.e. the functioning of healthcare systems) may influence people's attitudes towards self-diagnosis.

24.08.2023: Crystallisation Meeting - Coding

Over the past two weeks, Lucy and I have independently coded the same portion of the dataset. Today, we met to discuss our current set of codes and initial impressions of the data. As part of crystallisation, we aimed to discuss any similarities and differences in our interpretations to support me to continue analysis in a rich and considered way.

Firstly, I shared that my overarching take-away from data familiarisation was a sense of heaviness. Lucy commented that she had also felt this, and I found it interesting that we had both experienced a strong emotional response to the data. In particular, Lucy felt a sense of sadness from the data and this seemed to come from her reflection that well-intentioned attempts to destigmatise and raise awareness of mental disorders may be inadvertently increasing rates of inaccurate self-diagnosis, such that anyone using psychiatric language to communicate their distress is now met with scepticism. Lucy commented that the word 'despair' came to mind and this made me feel a sense of desperation - it brought to my mind the question: what can we do about this? If the situation regarding self-diagnosis is bad, how might we improve it? Will anyone offer a better alternative for understanding and expressing mental distress? This discussion broadened my reflections on the data from not just thinking about the nature of self-diagnosis and its possible consequences, but also what might we do about it.

Ultimately, we were both particularly receptive to the emotional tone of the data, or at least our own emotional responses to it. It seems that self-diagnosis is an emotionally charged and contentious topic.

Secondly, we had a particularly helpful discussion about the different possible forms of self-diagnosis. For example, we discussed the idea of tentative self-diagnosis (I think I have X)

and definite self-diagnosis (I know I have X). We also discussed how people commonly used the term 'fake' self-diagnosis and this could be interpreted in different ways, such as either being deliberately fake (i.e. actively pretending to have a mental disorder) or deluded (mistaking subclinical levels of distress as disorder). From this discussion, I've gained a sense of clarity over the different ways people have referred to self-diagnosis and I now feel more confident about turning towards analysis in a richer way by considering how attitudes towards self-diagnosis may depend on the form discussed.

08/09/2023: Coding

I'm now about halfway through coding - the second phase of analysis. I'm finding it quite overwhelming at times. The acceptability of SD seems to be based on a combination of intersecting factors and it feels hard to disentangle these during coding. For instance, acceptability seems to be based on (i) HOW someone is self-diagnosing (tentatively vs. with certainty, well-researched vs. using misinformation on social media), (ii) WHO is self-diagnosing (adolescents vs. adults), (iii) WHAT disorder they are diagnosing (e.g. DID seems to be particularly unacceptable), (iv) WHY they want to self-diagnose (e.g. to gain self-understanding in the face of genuine mental distress, or in attempt to gain social status), and (vi) WHO specifically is commenting on the acceptability of self-diagnosis (e.g. someone with a clinical diagnosis, someone who is undiagnosed but struggling with their mental health). In short, it's complex! It feels like there is an incredibly detailed (and exhausting) rulebook about when it is acceptable for someone to use diagnostic language to communicate their mental distress. One user made the following comment that seemed to sum this up: *'self-diagnosis can be a difficult thing to say is necessarily bad or good…it all depends on context'.*

I think theme development will require me to get clear on which of these factors are associated with particularly strong shared patterns of meaning across users.

29/09/2023: Condensing codes

I've finally finished coding and I seem to have reached a staggering number of codes: 450. However, over the past few days, I've been able to identify and remove duplicate codes and this has already reduced the number to 386. I think there was such a high number of duplicates because, throughout coding, I found it hard to keep track of the increasing number of codes when the dataset was saturated with such diverse and conflicting opinions.

My plan is to reduce the codes further, possibly to around 100-150, so that I have a manageable number for code generation. To do this, I'm going to firstly review the fairly hefty number of codes that only ended up with 1-2 related comments (about 50)– I plan to remove any codes where the associated data feels particularly shallow, not directly relevant to the research questions and/or can't be merged into an existing code. Then, I'm then going to work on merging similar codes and, where appropriate, create a set of higher-order codes with associated sub-codes. For example, I've formed the higher-order code *'people with a clinical diagnosis are not being taken seriously'* by merging existing codes into it as sub-codes, including *'inappropriate self-diagnosis takes attention away from those with a clinical diagnosis'*, *'inappropriate self-diagnosis increases stigma towards those with a clinical diagnosis'* and *'people with a clinical diagnosis aren't being believed'*.

Ultimately, I know that we won't be able to represent all the codes and the initially high number of codes may reflect a particularly fine-grained approach to analysis – given this, I might be able to merge codes relatively easily but I also needed to start letting go of ideas

that, whilst interesting, may not be directly relevant to the research questions. I hope I can reduce the codes down in a considered way, without losing any important concepts.

19/10/2023: Generating Initial Themes

I've now managed to reduce the codes to 90 and I have printed them off to provide a fresh perspective - away from NVivo and my laptop. I'm really enjoying working with the data in a more manual way - it feels much easier for me to take a broad overview of the dataset when I can lay the codes out on the floor and move them around in relation to one another. More recently, whilst generating themes, I have moved my laptop onto the floor (alongside the codes) so I can open NVivo and return to the raw data – to check that the way I'm interpreting the codes and generating themes is congruent with the raw data.

Several days ago, I particularly wanted to check the data associated with the code *'self-diagnosis can become self*-fulfilling', which I had provisionally decided to promote to a theme. This felt important because, whilst reflecting on my (ongoing) analysis, I recognised that I find this concept particularly interesting, and therefore during theme generation this code may have felt more salient to me relative to other codes. From the start of the project, I've found the concept that self-diagnosis may become self-fulfilling particularly interesting due to my personal and professional experiences. For instance, whilst working in a clinical health psychology service, I became particularly interested in the impact that language use may have on a person's physical wellbeing. Additionally, whilst recovering from chronic fatigue syndrome as a teenager, I had to relearn what normal levels of tiredness felt like and I noticed that whenever I misinterpreted normal levels of tiredness as fatigue or impending burnout, my distress increased and this exacerbated my symptoms. Given this, I recognised

that this code may have felt particularly salient to me, and this may have shaped my initial decision to promote it to a theme.

This awareness has prompted me to return to the data and become more thoughtful about this idea in relation to the entire dataset. I have also discussed my concern with Lucy. Having engaged in these forms of reflection, I actually feel more confident about shaping this code into a theme (*'self-diagnosis can become self-fulfilling'*); the code is relatively well-represented compared to other codes, and whilst some extracts seemed to make a relatively fleeting reference to the concept of self-diagnosis being self-fulfilling, others were particularly rich and insightful. Additionally, when I made the conscious effort to step back from my preexisting interests, there seemed to be a pressing sense of alarm and concern throughout this portion of the dataset. In this way, efforts to be reflexive has helped me engage in a particularly rich and considered analysis.

30/11/2023: Crystallisation meeting – Developing and Reviewing Themes

Yesterday, I met Lucy for a crystallisation meeting and we discussed my working themes. There were two themes that felt particularly helpful to discuss; exploring these together helped me consider broader possible perspectives on the data and acknowledge any assumptions that I may have brought to theme development.

Firstly, we discussed the theme: *'self-diagnosis is acceptable as a transitory theory'*. I shared that within this theme, I intended to capture that people most commonly believed that clinicians are the experts in diagnosis and therefore self-diagnosis should only be conducted in a tentative manner (I think I have X, rather than I know I have X), if at all. Lucy commented that whilst she had also noticed that most users seem to believe clinicians are the experts in diagnosis, some users appear to strongly believe that their lived experience and self-insight

gives them the upper hand in diagnosis. I had noticed this too - in fact, we had both highlighted this tension within our first crystallisation meeting - at the start of coding.

Despite appreciating this apparent tension, this discussion made me realise that I had been considering the question of ‘who is the expert in diagnosis, and how does this influence attitudes towards self-diagnosis?’ through the lens of what appeared to be the most common stance – viewing clinicians as experts. I wonder if I hadn’t immediately appreciated that the central organising concept of a theme could in fact be a tension within the data, not just a shared pattern of meaning. Ultimately, my discussion with Lucy made me revisit users’ broader opinions and acknowledge that despite being less represented, these opinions seemed unwavering and strongly expressed. In this way, the apparent tension around this topic became more salient to me. In response to this, I think I can provide richer insight into the data by expanding my original theme to represent users’ conflicting attitudes towards self-diagnosis rather than simply the most common stance. On reflection, I’ve realised that all along I’ve felt slightly unsettled about this theme – it didn’t feel quite right to present only the most common idea when there have been undercurrents of tension (e.g. self as expert, collaboration is preferable). Moving forwards, I’ve decided to expand my original theme to capture these insights from crystallisation and I’ve revised its name accordingly to *‘there is tension over who is the expert in diagnosis’*.

We also discussed the working theme *‘it’s awful with teenagers on social media’.* From early in analysis, Lucy and I had a strong sense that there was something important to represent about users’ views that adolescents are inaccurately self-diagnosing, often within the context of social media. However, within this meeting, we acknowledged that there appeared to be a slight difference in the finer details of our perspectives on this.

Specifically, some users provided clear reasons as to why adolescents may inaccurately self-diagnose (e.g. the way online misinformation interacts with adolescence-specific factors). In this way, these users appeared to highlight that although inappropriate, this behaviour is understandable. However, whilst I had been considering users' understanding as possibly conveying a sense of compassion towards adolescents, Lucy considered whether it might actually be used as a clear reason to dismiss them – it could be used as evidence to take their self-labelling even less seriously. It's interesting that we had interpreted this in different ways and perhaps the sentiment isn't always clear/explicitly stated in the comments - I think there are also extracts that support both our perspectives. Lucy shared that the overarching narrative that came to her mind was *'teenagers are to blame'*. In this way, Lucy appeared to view users' negative attitudes as a direct attack towards adolescents, rather than my impression of users conveying their frustration at the general situation and its contributing factors.

Returning to the data with an awareness of Lucy's perspective made me more thoughtful about the tone of the dataset and it made the ridicule directed at adolescents seem more salient. I wonder whether to some extent I have been blind to the intensity and widespread nature of the anger/ridicule directed at adolescents or maybe unintentionally underplaying this in some way - perhaps due to being an adolescent myself and experiencing confusing health challenges in my early teenage years. This may also explain why I was particularly touched by any possible sentiment of compassion towards young people who engage in self-diagnosis. Moving forward, I intend to take a more neutral and open-minded approach to extracts that outline why adolescents might self-diagnose inappropriately and consider their possible ambiguity in whether their sentiment is compassionate or dismissive.
