# Supplementary material for: Self-Diagnosis of Mental Disorders: A Qualitative Study of Attitudes on Reddit
Source: Qual Health Res. 2024 Oct 18;35(7):779–92. doi: 10.1177/10497323241288785 (PMC12056264; doi:10.1177/10497323241288785)
Supplement: Supplemental Material - Self-Diagnosis of Mental Disorders: A Qualitative Study of Attitudes on Reddit [file sj-pdf-2-qhr-10.1177_10497323241288785.pdf]

Supplementary Materials 2: Code List

In the initial coding of the dataset, the first author developed 450 codes. She then condensed and consolidated these to a list of 90 codes before theme generation. Listed below are the final 90 codes. The numbers in brackets indicates the prevalence of each code (i.e. the number of comments/phrases that were assigned that code). However, it should be noted that reflexive thematic analysis does not equate frequency with importance and given this, themes were primarily generated based on their relevance to the research questions (Braun & Clarke, 2022).

Braun, V., & Clarke, V. (2022). Conceptual and design thinking for thematic analysis. *Qualitative Psychology*, *9*(1), 3-26.

- Role (*i.e. the user indicated that they were clinically diagnosed with a mental disorder, struggling with mental health but had no clinical diagnosis, a healthcare professional, an adolescent, a parent, a teacher, etc;* 289)
- Self-diagnosis is understandable because getting professional support is hard (76)
- Clinicians are the experts in diagnosis (73)
- Self-diagnosis happens on social media/social media is to blame (59)
- Contrast between the severity of clinical diagnoses and presentations of self-diagnosis (51)
- Self-diagnosis is associated with adolescents – generally faking it (47)
- Self-diagnosing with certainty is not appropriate, tentative language is more acceptable (44)
- People with a clinical diagnosis are no longer being taken seriously (44)
- It is hard to self-diagnose in an objective, unbiased way (43)
- Self-diagnosis must be discussed with a healthcare professional (42)
- It is unacceptable to self-diagnose purely for ‘attention-seeking’ purposes (42)
- It is unacceptable when people self-diagnose in attempt to be cool/gain social status (41)
- It is unacceptable to use self-diagnosis as an excuse for undesirable behaviour (38)
- Self-diagnosis as a way to build a self-concept/self-identity (35)

- People are misinterpreting low-level distress and normal experiences as disorder (49)
- Psychiatric language is being overused (31)
- Inappropriate self-diagnosis takes attention and resources away from those who really need it (29)
- Can't always trust clinicians (28)
- Self-diagnosis as a starting point (27)
- People who self-diagnose are misinformed and deluded (26)
- Depression and anxiety disorders may be easier to accurately self-diagnose compared to personality disorders (26)
- Distinction – some self-diagnosis is genuine, some is fake (26)
- Self-diagnosis is a 'fake' diagnosis (25)
- Self-diagnosis can become a self-fulfilling prophecy (25)
- Self-diagnosis brings peace and self-understanding (24)
- Mental disorders are debilitating, not desirable (23)
- Well-researched self-diagnosis is acceptable (23)
- Inaccurate self-diagnosis can spread misinformation about mental disorders (20)
- Careless self-diagnosis is not acceptable (20)
- Self-diagnosing with a mental disorder has become more common (20)
- Identifying with a mental disorder may be encouraged/reinforced by others (20)
- 'Self' as expert in diagnosis (19)
- 'Fake' self-diagnosis (whether deliberate or deluded) can be a sign of distress in itself (18)
- Scepticism towards anyone claiming to have a mental disorder – is it real or fake (18)
- Strong negative language towards self-diagnosis (17)
- Inaccurate self-diagnosis may lead to inappropriate or unnecessary treatment (17)
- Living with a self-diagnosis can be hard – lack of support, despair (17)

- Collaboration between 'self' and clinician leads to the most accurate diagnosis (17)
- All distress deserves support, not just clinical levels (16)
- Self-diagnosing enables people to find social support and reduce sense of isolation (16)
- Identifying symptoms is acceptable and different from self-diagnosis (16)
- 'just trust them' – important to respect anyone claiming to have a mental disorder (16)
- Online communities that involve faking mental disorders are cult-like and manipulative (16)
- Self-diagnosis is inexcusably wrong – dismiss people who do it (15)
- Mental disorders still face stigma (15)
- Self-diagnosis should be private, not public (15)
- Deliberately faking mental disorders is unacceptable (15)
- Self-diagnosis can help people to self-manage symptoms, especially when professional support is out-of-reach (14)
- Self-diagnosis plays a fundamental/valuable role in healthcare interactions (14)
- Healthcare should be less about diagnostic labels and more about support (14)
- It is unacceptable for people to treat self-diagnosis as a fun game/hobby (14)
- Clinicians need to show that they understand a person's self-diagnosis whilst encouraging them to be open-minded (14)
- It can be hard to distinguish between low-level distress and signs of mental disorder (13)
- People holding mixed/conflicting attitudes towards self-diagnosis simultaneously (13)
- Need to encourage adolescents to express themselves and explore their identity through creative hobbies, not through inappropriate self-diagnosis (12)
- Inappropriate self-diagnosis means that genuine cases of self-diagnosis/distress are not being taken seriously (12)
- Expressing compassion and sensitivity towards adolescents who self-diagnose (12)
- People may become so attached to their self-diagnosis that they resist a clinician's differing opinion (12)

- Can’t trust self-diagnosis or clinical diagnosis (11)
- It can be hard to distinguish between typical adolescence and signs of mental disorder (10)
- Young people may self-diagnose because adolescence is a time of significant identity development and sensitivity to peer pressure (10)
- People want to feel prepared for their healthcare appointments (10)
- Teenagers feel like they aren’t being believed or taken seriously (10)
- Inaccurate self-diagnosis prevents people with a clinical diagnosis finding a supportive community (9)
- Some people ‘flaunt’ their self-diagnosis in a way that people with a clinical diagnosis/real mental disorder would not (9)
- Parents should do more to prevent their children from self-diagnosing inappropriately (9)
- Concern that disclosing self-diagnosis may bias a clinician’s judgement (8)
- Talking about symptoms is fundamental within healthcare interactions (8)
- Concern that clinicians may negatively judge people who self-diagnose (8)
- Self-diagnosis is a craving for an unmet need – love, attention, validation (8)
- Adolescents who identify with dissociative identity disorder are at risk of being groomed on social media (7)
- Self-diagnosis can be good – prompt people to seek support (7)
- Self-diagnosis is not the issue, misinformation is (7)
- Self-diagnosis is appealing because clinical diagnosis might lead to discrimination (7)
- Self-diagnosis is as (in)accurate as a clinical diagnosis (6)
- A better diagnostic system is needed (6)
- Stigma around mental disorders has reversed - mental illness is desirable and mental health is undesirable (6)
- Users’ accounts of when self-diagnosis did not match clinical judgment (6)

- Self-diagnosis and clinical diagnosis are both flawed but ultimately clinical diagnosis is better (6)
- Deliberately faking DID can become addictive (5)
- It is important to validate a person's self-diagnosis to encourage them to seek support (5)
- Adolescents are particularly vulnerable to inappropriately self-diagnose (5)
- 'Fake' self-diagnosis – there is a difference between 'deliberate' and 'deluded' (5)
- It is important that clinicians provide people who are inaccurately self-diagnosing with a framework of understanding that is helpful to that person (5)
- It is unacceptable to self-diagnose dissociative identity disorder because it is so rare (4)
- Adolescents will be embarrassed by their self-diagnosis in the future (4)
- Diagnostic language should be reserved for people with a clinical diagnosis (4)
- Self-identity and personality are inherently complex – people are unlikely to have dissociative identity disorder despite believing it (4)
- Need to work out how to address the high rates of self-diagnosed mental disorders (3)
- Increased rates of self-diagnosed mental disorders is a sign of reduced stigma (3)
