# Supplementary material for: Self-Diagnosis of Mental Disorders: A Qualitative Study of Attitudes on Reddit
Source: Qual Health Res. 2024 Oct 18;35(7):779–92. doi: 10.1177/10497323241288785 (PMC12056264; doi:10.1177/10497323241288785)
Supplement: Supplemental Material - Self-Diagnosis of Mental Disorders: A Qualitative Study of Attitudes on Reddit [file sj-pdf-3-qhr-10.1177_10497323241288785.pdf]

Supplementary materials 3. Additional methodology

Details regarding crystallisation process

Author 1 led the analysis, but both authors engaged in crystallisation together to deepen author 1's understanding of the data and wider research area and to support credible findings[43,44]. Specifically, the authors met throughout the analytical process to discuss similarities and differences in their interpretations of the data, with the aim of broadening RU's insights into its multiple possibilities and bringing awareness to her positionality. Notably, crystallisation is conceptually distinct from triangulation and aligns more closely with the authors' interpretivist, reflexive stance – whilst both practices involve bringing multiple perspectives to analysis, triangulation intends to search for a single valid truth, whereas crystallisation appreciates complexity within data and its multiple possible meanings[44]. Below, we highlight how crystallisation was used throughout the analysis.

After the second crystallisation meeting, in which the authors met to discuss the initial 450 codes, author 1 carried out a number of steps to produce a more manageable number of codes for the next phase of analysis. Similar codes were collated (e.g. the code *'people with a clinical diagnosis aren't being believed'* was moved into the existing code *'people with a clinical diagnosis are no longer being taken* seriously'); duplicate codes were merged; and some codes were discarded because they were associated with shallow, sparse data (e.g. the code *'clinical diagnosis feels permanent, self-diagnosis feels flexible'* was only mentioned briefly by two participants). These steps reduced the 450 initial codes to a list of 90 codes (see supplementary materials 2) and, based on these 90 codes, author 1 began the third phase of analysis, *theme generation*. Five preliminary themes were generated: *(1) Self-diagnosis is acceptable as a transitory theory; (2) Self-diagnosis is a route to self-*

understanding in a hostile system; (3) It's awful with teenagers on social media; (4) Self-diagnosis can become self-fulfilling and *(5) Now no one is believed*.

During the third crystallisation meeting, to review themes and propose theme names, several revisions being made. Firstly, the theme *'Self-diagnosis is acceptable as a transitory theory'* was expanded into a new theme *'There is tension over who is the expert in diagnosis'*. This enabled the authors to represent users' conflicting attitudes towards the acceptability of self-diagnosis, rather than simply conveying the most common attitude towards self-diagnosis, which was more descriptive. Secondly, the theme *'Self-diagnosis as a route to self-understanding in a hostile system'* was renamed *'Self-diagnosis is a route to self-understanding in an inaccessible system'* to highlight the central point that, for various reasons, many people are simply unable to access a diagnosis from a professional.

Thirdly, details of the theme *'It's awful with teenagers on social media'* were explored. The authors identified slight differences in their interpretation of data relating to this theme, specifically the extent to which users believed that teenagers are to blame or responsible for inappropriate self-diagnosis. By discussing multiple interpretations of the data, author 1 returned to analysis with a more reflexive stance and refined the theme to capture this ambiguity (see supplementary materials 1). The theme was also renamed *'Teenagers on social media are the problem'* to capture the more analytical perspective within this theme: that the interaction between young people and social media seemed to be the most central issue surrounding self-diagnosis.
